# Global response to physiotherapy services disruptions during the COVID-19 pandemic and the level of preparedness for the next health emergency

**DOI:** 10.3389/fresc.2025.1614604

**Published:** 2025-08-18

**Authors:** Heidi Kosakowski, Peter Skelton, Wouter De Groote, Jonathon Kruger, Flavio Salio

**Affiliations:** ^1^ World Physiotherapy, London, United Kingdom; ^2^ Department for Noncommunicable Diseases, Rehabilitation and Disability, World Health Organization, Geneva, Switzerland; ^3^ Department for Emergency Medical Teams, World Health Organization, Geneva, Switzerland

**Keywords:** physiotherapy, health emergency, rehabilitation, preparedness, planning

## Abstract

**Introduction:**

The COVID-19 pandemic was a global health emergency that severely impacted physiotherapy and other rehabilitation services. The purpose of this study is to describe mitigation strategies developed for physiotherapy service disruptions during the COVID-19 pandemic and the level of integration of physiotherapy services into health emergency preparedness planning in countries/territories of World Physiotherapy member organisations.

**Methods:**

The 2022 World Physiotherapy annual membership census (AMC) included questions on health emergency preparedness planning and mitigation strategies in line with WHO recommendations. Quantitative analysis was conducted on response frequencies and disaggregated into World Physiotherapy regions and country/territory income level classifications.

**Results:**

116 out of 125 World Physiotherapy member organisations (MOs) participated in the census. 24% of all participating MOs reported not adopting any of the listed mitigation strategies to overcome physiotherapy service disruptions in their country/territory during the COVID-19 pandemic. 64% of participating MOs reported that physiotherapy services were not included in any health emergency preparedness component for rehabilitation in their country/territory.

**Discussion:**

There are low levels of integration of physiotherapy services into national and subnational health emergency preparedness planning. A country's income level does not appear to be a major determinant of emergency preparedness.

## Introduction

Health emergencies, whether due to disaster, war or disease outbreak, create enormous surges in acute and long-term rehabilitation needs while also simultaneously disrupting essential rehabilitation services (1). Rehabilitation has increasingly been recognized as an essential health service and integral to strong health systems. Awareness is increasing regarding rehabilitation as an essential health service in emergencies that plays a critical role in determining patient outcomes (2, 3). However, rehabilitation services are commonly among the most disrupted services and are often late to be considered as part of any response, if indeed they are considered at all (4).

The COVID-19 pandemic was a global health emergency, impacting all countries around the world. Rehabilitation services formed an essential part of acute clinical care, post-acute follow up, and longer-term rehabilitation for the Post COVID-19 condition (Long COVID). However, rehabilitation services were also amongst the most severely disrupted by the pandemic (5–7).

Key to improving rehabilitation responses is a greater emphasis on rehabilitation as an integral component of health emergency preparedness (4). The integration of rehabilitation into all-hazard preparedness is mandated by many international conventions (8, 9), and is specifically called for in World Health Assembly Resolution 76.6 (10). There is little available evidence of the extent to which rehabilitation services are currently integrated into countries' emergency preparedness. Three examples exist in the literature—in Japan (11), Nepal (12) and Iran (13). Experiences from a wide range of recent emergencies in low-, middle-, and high-income settings indicate that rehabilitation preparedness is an exception and not a norm. Therefore, it is vital to start describing the extent to which rehabilitation services have been integrated into national or subnational health emergency preparedness planning around the world.

The focus of this paper is on physiotherapy services, as an integral part of rehabilitation, due to the available data and methodology used. Physiotherapy is one of several service types that compromise rehabilitation services.

World Physiotherapy is the sole global organisation representing more than 600,000 physiotherapists through their national professional physiotherapy association. It has been a non-state actor in official relations with the World Health Organization (WHO) since 1956.

Every year World Physiotherapy sends an annual membership census (AMC) to each of its member organisations (MO) with questions on membership, physiotherapy workforce, scope of practice, education, and regulation, among others. The information collected in the AMC is used to create a profile of the profession and national organisation (14). Further, national data is aggregated into regional and global reports. MOs can use the profiles to compare the status of physiotherapy in their country/territory with that of their region and globally, identifying gaps and areas in need of strengthening, and then use the information as an advocacy tool with their national governments.

The goal of this study was to use AMC data to describe mitigation strategies developed for physiotherapy service disruptions during the COVID-19 pandemic and the level of integration of physiotherapy services into health emergency preparedness planning in countries/territories of World Physiotherapy MOs.

## Materials and methods

### Research questions

The questions that guide this study are:

1. Regarding mitigation strategies to respond to disruptions in physiotherapy services during the COVID-19 pandemic, what strategies have been developed and what association exists between the mitigation strategies developed and the country income level of World Physiotherapy member organisations?
2. With respect to health emergency preparedness planning in countries/territories of World Physiotherapy member organisations, how have physiotherapy services been integrated and what association exists between the country income level and geographical location and the level of integration?

### Annual membership census (AMC)

**Figure 1:**
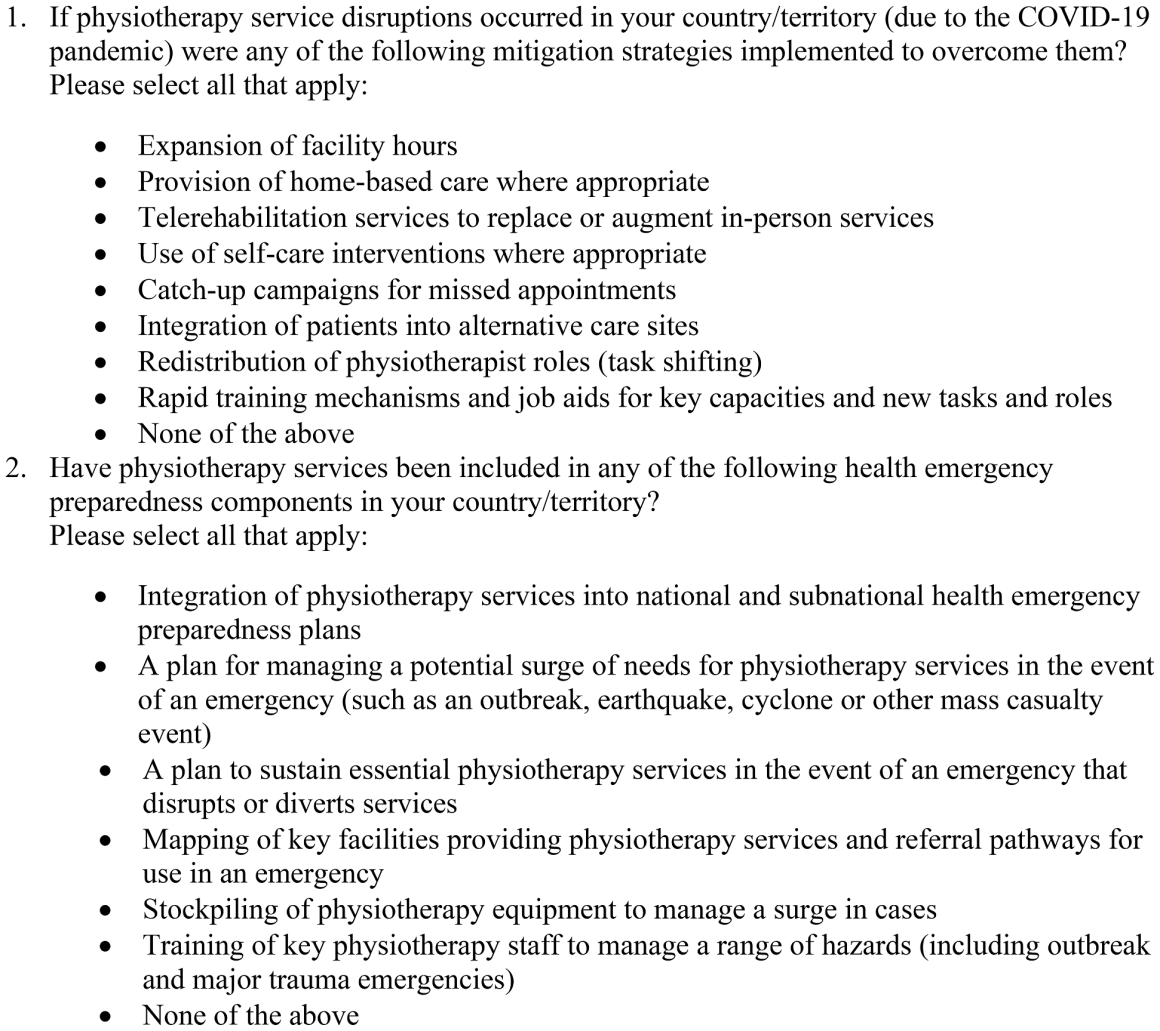
World Physiotherapy AMC questions on health emergency response and preparedness.

The 2022 annual membership census (AMC) of World Physiotherapy was adapted to include additional questions for answering our research questions. In addition to the standard AMC, two multiple choice questions on health emergency response and preparedness were developed (Figure 1). The first question was adapted from the WHO global pulse survey on continuity of essential health services during the COVID-19 pandemic containing similar mitigation strategies for selection (5). The second question was developed with the WHO Rehabilitation Programme and based on core components for health emergency preparedness planning. The response options for the second question contain similar components to the WHO indicator on health emergency preparedness for rehabilitation (15), which contain 8 components and was published after the AMC questions were developed.

Respondents were permitted to select more than one response option for each question. For question 2, an open field box was available to provide more information on any health emergency preparedness component.

AMC questions have not been piloted or cognitively tested prior to inclusion in the census. Questions were written in English and translated into French and Spanish by the authors and checked for conveying the same questions as in the original English version by colleagues that were native French and Spanish speakers. Respondents representing MOs were instructed to contact World Physiotherapy if they needed clarification on any AMC questions.

### Participants

As per standard procedures, the AMC was sent to the primary contact for each World Physiotherapy MO. World Physiotherapy has only one MO per country/territory. Member organisations are divided into 5 World Physiotherapy regions: Africa, Asia Western Pacific region (AWP), Europe, North America Caribbean region (NACR), and South America region (SAR). Responses to AMC questions may be based on authoritative national data sources or informal sources of information from individuals with deep knowledge of the profession.

### Data analysis

Descriptive statistical analyses were conducted for response frequencies on each multiple-choice question on health emergency response and preparedness using Microsoft Excel. Frequencies for each response option were calculated for all participating countries/territories, each region, and by country/territory income level.

Two chi-square tests of independence were conducted. A chi-square test of independence was conducted to examine the relationship between country/territory income level and health emergency components. Another chi-square test of independence was conducted to examine the relationship between regions and mitigation strategies.

## Results

In September 2022, almost 2.5 years into the COVID-19 pandemic, World Physiotherapy sent its AMC with additional questions to 125 MOs. 116 of 125 MOs completed and returned the AMC to World Physiotherapy: 23 MOs from the Africa region, 28 from AWP, 41 from Europe, 14 from NACR, and 10 from SAR. 78 of the MOs are in high and upper-middle income countries/territories, 27 are in middle and lower-middle income countries/territories, and 11 are in low-income countries/territories.

No MO contacted World Physiotherapy for clarification on the AMC questions pertaining to COVID-19 mitigation strategies or the inclusion of physiotherapy in health emergency preparedness.

### Mitigation strategies

24% of all participating MOs reported not adopting any of the listed mitigation strategies to overcome physiotherapy service disruptions in their country/territory during the COVID-19 pandemic. Of the 76% of MOs that reported having mitigation strategies, the most frequently cited strategy was telerehabilitation (57%) to replace or augment in-person services; followed by 42% for provision of home-based care where appropriate; 41% for promotion of self-care interventions where appropriate; 39% for redistribution of physiotherapist roles (task shifting); 24% for an expansion of facility hours; 19% for rapid training mechanisms and job aids for key capacities and new tasks and roles; 12% for integration of patients into alternative care sites; and 9% for catch-up campaigns for missed appointments. 65% of all respondents reported more than one mitigation strategy.

Telerehabilitation was the most frequently reported mitigation strategy in all regions except the Africa region, which reported home-based care as the most frequent mitigation strategy (Table 1).

**Table 1:** Percentage of countries/territories with mitigation strategies developed to overcome physiotherapy service disruptions, by World Physiotherapy region.

Mitigation strategies	Africa (*N* = 23)	AWP (*N* = 28)	Europe (*N* = 41)	NACR (*N* = 14)	SAR (*N* = 10)	All regions (*N* = 116)
Hours expansion	26% (6)	25% (7)	22% (9)	21% (3)	30% (3)	24% (28)
Home-based care	48% (11)	43% (12)	27% (11)	64% (9)	60% (6)	42% (49)
Telerehabilitation	26% (6)	57% (16)	59% (24)	79% (11)	90% (9)	57% (66)
Self-care	30% (7)	46% (13)	39% (16)	50% (7)	40% (4)	41% (47)
Catch up campaigns	9% (2)	18% (5)	2% (1)	21% (3)	0	9% (11)
Alternative care	13% (3)	14% (4)	2% (1)	36% (5)	10% (1)	(14)12%
Task shifting	35% (8)	36% (10)	39% (16)	36% (5)	60% (6)	39% (45)
Rapid training	17% (4)	14% (4)	24% (10)	14% (2)	20% (2)	19% (22)
More than 1	56% (13)	54% (15)	66% (27)	86% (12)	80% (8)	65% (75)
No strategy	30% (7)	25% (7)	27% (11)	14% (2)	10% (1)	24% (28)

The result of the chi-square test found no association between region and mitigation strategies (*p*-value 0.65; chi-square statistic: 28.47, degrees of freedom: 32).

### Health emergency preparedness components

64% of participating MOs (74/116) reported that physiotherapy services were not included in any health emergency preparedness component in their country/territory. The remaining 36% MOs (42/116) reported that physiotherapy was integrated into one or more health emergency preparedness components. The most frequently cited component was training key physiotherapy staff to manage a range of hazards at 18% (21/116); followed by 17% (20/116) for integration of physiotherapy into national and subnational health emergency preparedness plans; 15% (17/116) for having a plan for managing a potential surge of physiotherapy needs in the event of an emergency; 12% (14/116) for having a plan to sustain essential physiotherapy services in the event of an emergency that disrupts or diverts services; 11% (13/116) for mapping of key facilities providing physiotherapy and referral pathways for use in an emergency; and 8% (9/116) for stockpiling of physiotherapy equipment to manage a surge in rehabilitation cases (Table 2).

**Table 2:** Percentage of countries/territories that have physiotherapy services integrated in health emergency preparedness components, by World Physiotherapy region.

Emergency preparedness component	Africa (*N* = 23)	AWP (*N* = 28)	Europe (*N* = 41)	NACR (*N* = 14)	SAR (*N* = 10)	All regions (*N* = 116)
Integration	17% (4)	25% (7)	12% (5)	14% (2)	20% (2)	17% (20)
Surge plan	9% (2)	32% (9)	7% (3)	14% (2)	10% (1)	15% (17)
Continuity plan	9% (2)	14% (4)	10% (4)	21% (3)	10% (1)	12% (14)
Mapping	13% (3)	14% (4)	7% (3)	14% (2)	10% (1)	11% (13)
Stockpiling	0	7% (2)	12% (5)	7% (1)	10% (1)	8% (9)
Training	13% (3)	25% (7)	15% (6)	21% (3)	20% (2)	18% (21)
More than one	13% (3)	25% (7)	17% (7)	14% (2)	10% (1)	17% (20)
No component	74% (17)	54% (15)	68% (28)	50% (7)	70% (7)	64% (74)

**Figure 2:**
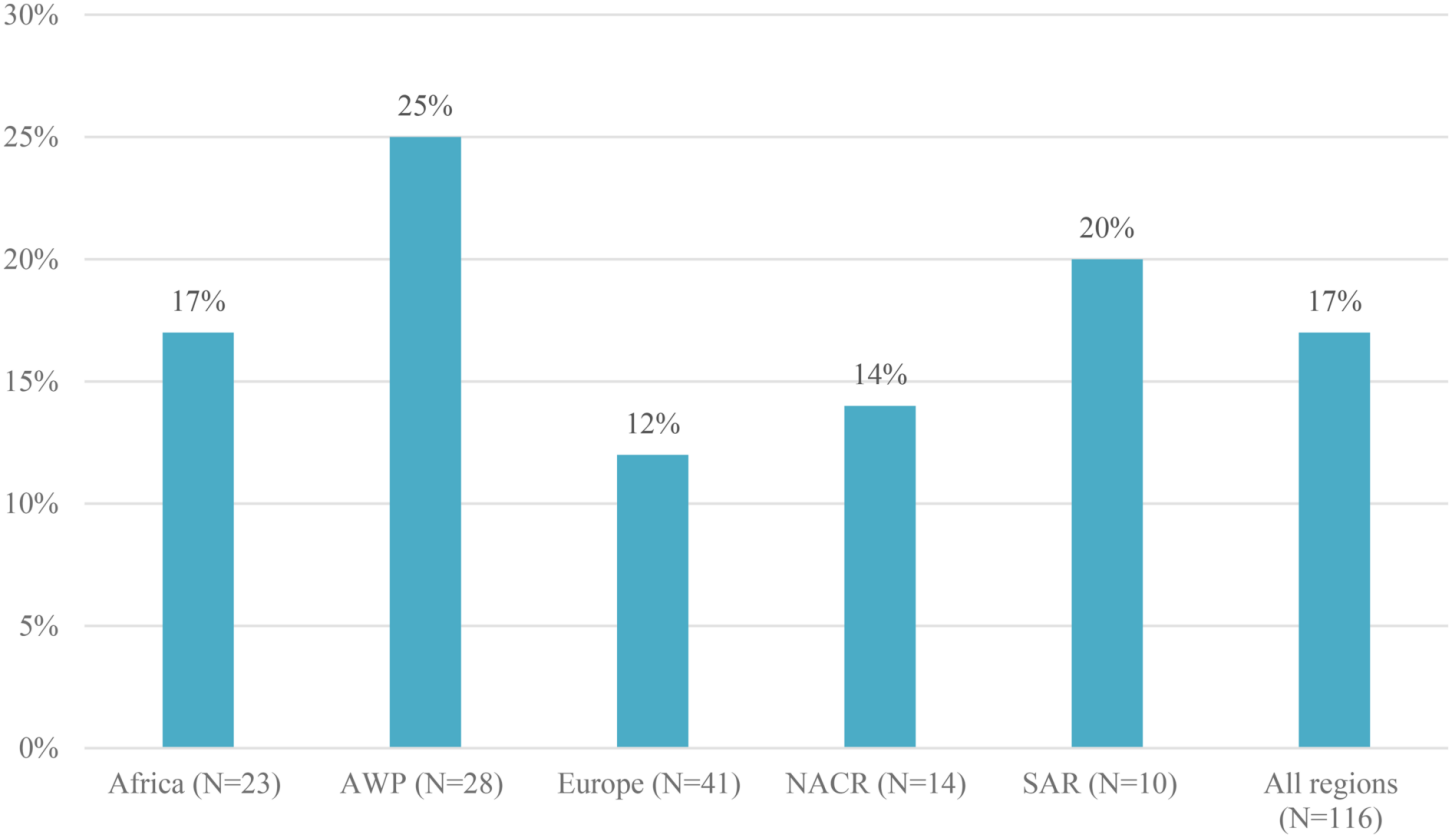
Percentage of countries/territories with physiotherapy services integrated into national and subnational health emergency preparedness plans.

17% of MOs (20/116) reported having included physiotherapy in more than one health emergency preparedness component in their country/territory (Table 2 and Figure 2); 3 of the 23 MOs in the Africa region, 7 of the 28 MOs in AWP, 7 of the 41 MOs in the Europe region, 2 of the 14 MOs in NACR, and only 1 of the 10 MOs in SAR. Integration of physiotherapy services into (sub)national health emergency preparedness plans was reported in all 3 MOs in the Africa region (Ethiopia, Kenya, Togo) that selected more than one component. In AWP, surge planning and integration of physiotherapy into (sub)national health emergency preparedness plans were most reported in Australia, Fiji, India, New Zealand, Philippines, Saudi Arabia and Yemen. In the Europe region, integration into (sub)national health emergency preparedness plans, stockpiling physiotherapy equipment, and training of key physiotherapy staff were identified as the most frequent components when multiple components were selected in Belgium, Bosnia and Herzegovina, Denmark, France, Palestine, Poland, Slovakia and the United Kingdom. In NACR, having a plan to sustain essential physiotherapy services in the event of an emergency, and mapping of key facilities providing physiotherapy services and referral pathways for use in an emergency were selected for both Guyana and Jamaica. In SAR, Costa Rica reported the inclusion of physiotherapy in all health emergency preparedness components.

In terms of absolute numbers of components for health emergency preparedness planning that included physiotherapy services, 8% (9) of countries have 2 components, 3% (3) of countries have 3 components, 2% (2) of countries have 4 components, 3% (3) of countries have 5 components, and 3% (4) of countries have all 6 components (Table 3).

**Table 3:** Countries/territories and number of components, by World Physiotherapy region.

Africa	AWP	Europe	NAC	SAR
Ethiopia (5)	Australia (2)	Austria (1)	Barbados (1)	Brazil (1)
Ghana (1)	Bangladesh (1)	Belgium (3)	Bermuda (1)	Chile (1)
Kenya (2)	Bhutan (1)	Bosnia and Herzegovina (2)	Guyana (5)	Costa Rica (6)
Mauritius (1)	Fiji (6)	Czech Republic (1)	Jamaica (3)	
Togo (4)	India (5)	Denmark (2)	Panama (1)	
Uganda (1)	Indonesia (1)	France (2)	Puerto Rico (1)	
	Japan (1)	Hungary (1)	St Lucia (1)	
	Kuwait (1)	Latvia (1)		
	Nepal (1)	Malta (1)		
	New Zealand (4)	Palestine (2)		
	Philippines (2)	Poland (2)		
	Saudi Arabia (6)	Slovakia (3)		
	Yemen (2)	United Kingdom (6)		

**Figure 3:**
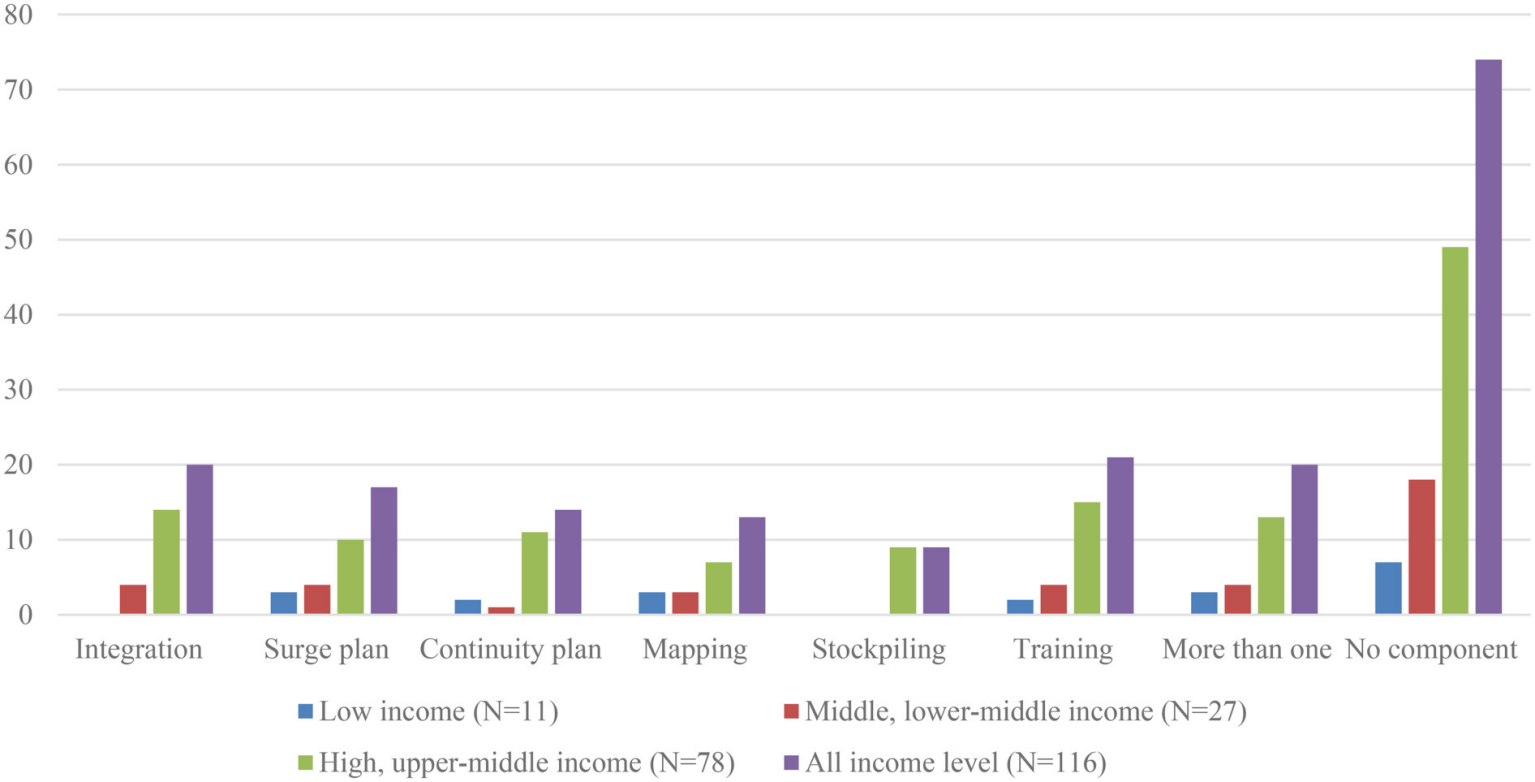
Health emergency preparedness components by income level.

The most frequently cited health emergency preparedness planning component in high and upper-middle income countries/territories was training of key physiotherapy staff to manage a range of hazards (19%) (Table 4, Figure 3). Training of key physiotherapy staff to manage a range of hazards and integration of physiotherapy services into national and subnational health emergency preparedness plans were equally reported as the most frequent health emergency preparedness component in upper-middle (19%), lower-middle (15%) and low income (18%) countries/territories (Table 4).

**Table 4:** Percentage of countries/territories that have physiotherapy services integrated in health emergency preparedness components, by country income level.

Emergency preparedness component	Low income (*N* = 11)	Middle, lower-middle income (*N* = 27)	High, upper-middle income (*N* = 78)	All income level (*N* = 116)
Integration	18% (2)	15% (4)	18% (14)	17% (20)
Surge plan	27% (3)	15% (4)	13% (10)	15% (17)
Continuity plan	18% (2)	4% (1)	14% (11)	12% (14)
Mapping	27% (3)	11% (3)	9% (7)	11% (13)
Stockpiling	0	0	12% (9)	8% (9)
Training	18% (2)	15% (4)	19% (15)	18% (21)
More than one	27% (3)	15% (4)	17% (13)	17% (20)
No component	64% (7)	67% (18)	63% (49)	64% (74)

The result of the chi-square test found no association between income level and health emergency preparedness measures (*p*-value 0.98, chi-square statistic:4.13, degrees of freedom: 12).

## Discussion

Our study aimed to assess mitigation strategies to respond to disruptions in physiotherapy services during the COVID-19 pandemic and the level of preparedness for a next health emergency in countries/territories of World Physiotherapy MOs. For this, an AMC with 2 additional questions has been completed by 116/125 MOs of World Physiotherapy at the end of 2022 during the third year of the COVID-19 pandemic. 64% of participating MOs reported that physiotherapy services were not included in any health emergency preparedness component for rehabilitation in their country/territory. Despite limitations in preparedness, a wide range of mitigation strategies were adopted during the COVID-19 pandemic. Further research, awareness raising, and advocacy are needed to increase the percentage of countries/territories where physiotherapy and other rehabilitation services are integrated into health emergency preparedness planning and mitigation strategies. Technical support from international organizations and national governments is critical to this advancement.

### Mitigation strategies

In terms of mitigation strategies, a key finding of this study is the high percentage of World Physiotherapy MOs that reported mitigation strategies to overcome physiotherapy service disruptions in their country/territory. While this was due to a response to the COVID-19 pandemic, the strategies could be used in other emergency contexts. With no relationship between World Physiotherapy regions and mitigation strategies, the most frequently reported mitigation strategies for physiotherapy services are similar to those reported in the global WHO Pulse Survey 2023 (16). Both AMC respondents and WHO member states representing multiple health sectors reported telerehabilitation/telemedicine, home-based care, and use of self-care interventions among the top strategies implemented in response to the COVID-19 pandemic.

The most commonly adopted mitigation strategy was telerehabilitation to replace or augment in-person services. While telerehabilitation may be a key mitigation strategy, particularly for service disruption due to a pandemic, considerations should be made regarding appropriate device availability and connectivity for future use. Challenges with these likely contributed to the low uptake of telerehabilitation in the Africa region which has the highest percentage of low-income countries and had the lowest telerehabilitation uptake compared with other regions. Moreover, while delivering physiotherapy services via telerehabilitation has evidence to support its effectiveness, it should not be seen as a solution to all emergency related challenges as patient, physiotherapist, and organizational barriers are known to exist (17). It cannot replace inpatient acute and post-acute rehabilitation and is unlikely to be helpful in managing the acute rehabilitation needs created by many emergencies.

Many of the mitigation steps adopted, including task shifting, the formal promotion of self-care, rapid training, expanded service hours and identification of alternative care sites are also critical steps to manage a surge and must be included in emergency preparedness plans to be able to be safely and rapidly implemented.

#### Preparedness measures

A key finding is the low level of integration of physiotherapy services into national and subnational health emergency preparedness plans (17%), although 36% of MOs (42/116) reported that one or more preparedness measures had been taken by their members. This was, however, rarely comprehensive, as is seen in the low percentages of MOs that reported having more than one health emergency preparedness component in place. Regardless of profession specific steps taken by member organizations, the lack of formal integration of rehabilitation into national or sub-national preparedness is likely to be a major barrier to any rapid and effective response.

The association between country/territory income level and health emergency preparedness measures was not found to be statistically significant, suggesting that income level was not associated with the likelihood of adopting specific emergency preparedness measures during the reporting period. Based on this finding, the authors recommend that countries/territories in all income classifications work towards implementing comprehensive preparedness measures.

This study provides baseline information on the extent to which physiotherapy services are part of health emergency preparedness planning and can be used to set goals using the WHO indicator on health emergency preparedness for rehabilitation as a benchmark. The WHO indicator on health emergency preparedness for rehabilitation describes 8 key components that should be included in health emergency planning (15) and are very much like the components assessed with the AMC. World Physiotherapy MOs are positioned to advocate to their ministries of health for physiotherapy and rehabilitation in (sub)national health emergency planning.

Any differences noted in the frequency of preparedness measures may be of relevance to planners when seeking to implement preparedness measures in their local context. For example, the absence of stockpiling of equipment may be due to limited resources available (where existing assistive product needs may already be unmet (18), and alternative methods of ensuring a surge in the supply of essential products such as crutches and wheelchairs should be identified. The lower use of service mapping as a specific preparedness measure may be due to strength of existing networks and referral pathways in such settings, whereas where rehabilitation services are weaker, more poorly integrated into the health system or fragmented, greater mapping is often needed.

Our findings support previous research (19–21) that has highlighted the low awareness (both inside and outside of the workforce) of the role of rehabilitation in emergencies—which is a major barrier to its inclusion in preparedness. Other barriers may include a lack of leadership in this area while the historical lack of an agreed preparedness framework for rehabilitation may have contributed to the wide variation in measures taken.

To address these gaps, more awareness raising needs to occur via advocacy on the inclusion of rehabilitation services into national and subnational planning. Several policy and advocacy tools now exist to support this, such as the WHO policy brief on Strengthening rehabilitation in health emergency preparedness, readiness, response and resilience (4), the World Physiotherapy policy statement on disaster management (22), the landmark World Health Assembly resolution 76.6 on Strengthening rehabilitation in health systems (10), and resources developed by the World Rehabilitation Alliance emergency workstream (23). In addition, the latter is in a good position to develop a systemic approach to support country advocacy activities. Furthermore, technical support to countries for better including rehabilitation into health emergency preparedness is needed, and WHO is currently developing a practical framework and toolkit to support rehabilitation preparedness.

### Future research

Future World Physiotherapy AMCs should continue to include questions on mitigation strategies and preparedness measures. Longitudinal analyses should be conducted to monitor change from the baseline provided in this study. This should be complemented with evidence of integration into (sub)national planning and context-specific qualitative information and detailed examples of mitigation strategies and preparedness measures.

Other international rehabilitation organizations should consider developing similar surveys aimed at understanding mitigation strategies and the level of integration of other rehabilitation professions at the (sub)national level. The WHO indicator for health emergency preparedness for rehabilitation can be used to monitor preparedness.

Future studies on mitigation strategies and health emergency preparedness measures in rehabilitation should include a comparison of disaster risk to level of preparedness and document effective strategies and measures.

### Limitations

Validation of data was not confirmed with ministries of health. Responses to the AMC were provided by MO representatives who may not work in a ministry of health or have knowledge of recent or ongoing plans to include rehabilitation into preparedness planning at the government level. As a member's survey, there is also the potential for an advocacy bias in responses.

Response options in the AMC did not allow for in-depth reporting of information regarding the quantity or quality of mitigation strategies or preparedness measures in each country/territory. AMC respondents were not asked to provide data sources or examples of mitigation strategies or preparedness measures for verification.

Further, how well MOs understood the AMC questions is unknown. AMC questions were not cognitively tested, nor was a definition of mitigation strategies or preparedness measures provided. Responses are therefore interpretative of the respondent, and the descriptive nature of the study precludes causal interpretation.

## Data Availability

The datasets presented in this article are not readily available. Requests to access the datasets should be directed to hkosakowski@world.physio.
